# Live, Attenuated Influenza A H5N1 Candidate Vaccines Provide Broad Cross-Protection in Mice and Ferrets

**DOI:** 10.1371/journal.pmed.0030360

**Published:** 2006-09-12

**Authors:** Amorsolo L Suguitan, Josephine McAuliffe, Kimberly L Mills, Hong Jin, Greg Duke, Bin Lu, Catherine J Luke, Brian Murphy, David E Swayne, George Kemble, Kanta Subbarao

**Affiliations:** 1 Laboratory of Infectious Diseases, National Institute of Allergy and Infectious Diseases, National Institutes of Health, Bethesda, Maryland, United States of America; 2 MedImmune Vaccines, Mountain View, California, United States of America; 3 Southeast Poultry Research Laboratory, Agricultural Research Service, United States Department of Agriculture, Athens, Georgia, United States of America; The University of Hong Kong, China

## Abstract

**Background:**

Recent outbreaks of highly pathogenic influenza A H5N1 viruses in humans and avian species that began in Asia and have spread to other continents underscore an urgent need to develop vaccines that would protect the human population in the event of a pandemic.

**Methods and Findings:**

Live, attenuated candidate vaccines possessing genes encoding a modified H5 hemagglutinin (HA) and a wild-type *(wt)* N1 neuraminidase from influenza A H5N1 viruses isolated in Hong Kong and Vietnam in 1997, 2003, and 2004, and remaining gene segments derived from the cold-adapted *(ca)* influenza A vaccine donor strain, influenza A/Ann Arbor/6/60 *ca* (H2N2), were generated by reverse genetics. The H5N1 *ca* vaccine viruses required trypsin for efficient growth in vitro, as predicted by the modification engineered in the gene encoding the HA, and possessed the temperature-sensitive and attenuation phenotypes specified by the internal protein genes of the *ca* vaccine donor strain. More importantly, the candidate vaccines were immunogenic in mice. Four weeks after receiving a single dose of 10^6^ 50% tissue culture infectious doses of intranasally administered vaccines, mice were fully protected from lethality following challenge with homologous and antigenically distinct heterologous *wt* H5N1 viruses from different genetic sublineages (clades 1, 2, and 3) that were isolated in Asia between 1997 and 2005. Four weeks after receiving two doses of the vaccines, mice and ferrets were fully protected against pulmonary replication of homologous and heterologous *wt* H5N1 viruses.

**Conclusions:**

The promising findings in these preclinical studies of safety, immunogenicity, and efficacy of the H5N1 *ca* vaccines against antigenically diverse H5N1 vaccines provide support for their careful evaluation in Phase 1 clinical trials in humans.

## Abstract 2

Promising preclinical results on safety, immunogenicity, and efficacy against diverse H5N1 strains provide support for careful evaluation of live, attenuated H5N1 vaccines in clinical trials in humans.

## Editors' Summary

**Background.:**

Influenza A viruses are classified into subtypes according to two of the proteins from the virus surface, the hemagglutinin (HA) and neuraminidase (NA) proteins, each of which occurs naturally in several different versions. For example, the global epidemic (pandemic) of 1918–1919 was caused by an influenza virus containing subtype 1 hemagglutinin and subtype 1 neuraminidase (H1N1), the 1957–1958 pandemic involved an H2N2 virus, and the 1969 pandemic, H3N2. Since 1997, several serious outbreaks of H5N1 infection have occurred in poultry and in humans, raising concerns that H5N1 “bird flu” may cause the next human influenza pandemic. Although human-to-human transmission of H5N1 viruses appears limited, mortality rates in human outbreaks of the disease have been alarmingly high—approximately 50%. A protective vaccine against H5N1 influenza might not only benefit regions where transmission from poultry to humans occurs, but could conceivably avert global catastrophe in the event that H5N1 evolves such that human-to-human spread becomes more frequent.

**Why Was This Study Done?:**

Several approaches are in progress to develop vaccines against H5N1 viruses. To date, the products that have been tested in humans have not been very effective in producing a strong immune response. To be optimal for human use, a vaccine would have to be very safe, remain stable in storage, and provide protection against influenza caused by naturally occurring H5N1 viruses that are not precisely identical to the ones used to make the vaccine. This study was done to develop a new H5N1 vaccine and to test it in animals.

**What Did the Researchers Do and Find?:**

The researchers developed vaccines using three artificially constructed, weakened forms of the H5N1 influenza virus. The three vaccine viruses were constructed using flu virus proteins other than HA and NA from an artificially weakened (attenuated) strain of influenza. These were combined in laboratory-grown cells with HA and NA proteins from H5N1 viruses isolated from human cases during three different years: 2004, 2003, and 1997. They grew larger quantities of the resulting viruses in hen's eggs, and tested the vaccines in chickens, ferrets, and mice.

In tests of safety, the study found that, unlike the natural viruses from which they were derived, the vaccine strains did not cause death when injected into the bloodstream of chickens, and did not even cause infection when given through the birds' breathing passages. Similarly, while the natural viruses were lethal in mice at various doses, the vaccine strains did not cause death even at the highest dose. In ferrets, infection with the vaccine strains was limited to the upper respiratory tract, while the natural viruses spread to the lungs and other organs.

In tests of protection, all mice that had received any of the three vaccines survived following infection with any of the natural viruses (so-called viral challenge), while unvaccinated mice died following viral challenge. This occurred even though standard blood tests could not detect a strong immune responses following a single dose of vaccine. Challenge virus was detected in the lungs of the immunized mice, but at lower levels than in the unvaccinated mice. Mice given two doses of a vaccine showed stronger immunity on blood tests, and almost complete protection from respiratory infection following challenge. In addition, mice and ferrets that had received two doses of vaccine were protected against challenge with H5N1 strains from more recent outbreaks in Asia that differed substantially from the strains that were used for the vaccine.

**What Do These Findings Mean?:**

This study shows that it is possible to create a live, attenuated vaccine based on a single H5N1 virus that can provide protection (in mice and ferrets, at least) against different H5N1 viruses that emerge years later. Attenuated influenza virus vaccines of this sort are unlikely to be useful to protect fowl because they do not infect or induce an immune response in chickens. However, while the safety and protection found in small animals are encouraging, it is not possible to know without human testing whether a vaccine that protects mice and ferrets will work in humans, or how this type of vaccine may compare with others being developed for use in humans against H5N1 influenza. Tests of one of the vaccines in human volunteers in carefully conducted clinical trials are currently under way.

**Additional Information.:**

Please access these Web sites via the online version of this summary at http://dx.doi.org/10.1371/journal.pmed.0030360.

- WHO Influenza Pandemic Preparedness page
- US Department of Health and Human Services Avian and Pandemic flu information site
- Wikipedia entry on H5N1 (note: Wikipedia is a free Internet encyclopedia that anyone can edit)
- CDC Avian Influenza Web page

## Introduction

On several occasions since 1997, outbreaks of highly pathogenic avian influenza (HPAI) H5N1 infections have occurred in poultry and in humans, fueling public health concerns over their potential to ignite a pandemic that would cause significant morbidity and loss of life. The most recent H5N1 outbreak in poultry, which began in late 2003, affected at least ten Asian countries initially, but recent reports indicate that H5N1 viruses have been isolated in wild birds and poultry in several countries in Asia, Europe, and Africa. More importantly, human cases of H5N1 infections have been reported since December 2003 in nine countries, with a total of 194 laboratory-confirmed cases and 109 fatalities as of 12 April 2006 (http://www.who.int/csr/disease/avian_influenza/country/cases_table_2006_04_12/en/). Although antiviral drugs are valuable for the prevention and treatment of H5N1 infections, they are limited in supply; in addition, resistance to these antivirals—which include drugs that target neuraminidase (NA) as well as those that block M2 ion channel function—has been described in multiple isolates [1–3]. The development of an H5N1 vaccine is recognized as the primary strategy to protect humans against a possible H5N1 pandemic. Infections with H5N1 influenza viruses in avian species and in humans have been occurring sporadically since 1997 and phylogenetic and antigenic analyses of H5N1 viruses collected over this period indicate that they have evolved into different sublineages or clades: 2004 viruses are designated clade 1, 2003 viruses clade 1′, some 2005 isolates clade 2, and the 1997 viruses clade 3 [4,5]. Antigenic differences among influenza viruses are usually identified by analysis of specific postinfection ferret antisera in reciprocal hemagglutination-inhibition (HAI) assays. Reports of genetic and antigenic changes observed in the H5N1 viruses isolated between 1997 and 2005 have raised concern about the ability of a vaccine generated from a single selected strain to protect against a diverse set of viruses [5,6].

The two modalities that are licensed as vaccines in the U.S. against human influenza viruses are inactivated split virus and live, attenuated virus vaccines. The development of effective vaccines against HPAI H5N1 viruses, however, presents several challenges. Due to their high pathogenicity, handling of the wild-type *(wt)* viruses requires biosafety level 3 (BSL-3) containment. Moreover, these viruses are highly pathogenic in chickens and are also lethal for chick embryos, so they cannot be propagated efficiently and safely in eggs. Several strategies have been explored in attempts to overcome these obstacles. These include the use of a low-pathogenicity H5N3 avian influenza virus that is antigenically related to circulating strains [7], the use of recombinant H5 hemagglutinin (HA) expressed in baculovirus [8], the use of recombinant adenoviruses expressing the H5 HA [9,10], and the production of attenuated seed viruses with the H5 HA modified by means of reverse genetics [11,12].

Nicholson and colleagues [7] investigated the feasibility of using an antigenically related, low-pathogenicity H5N3 influenza virus (influenza A/duck/Singapore/97) inactivated virus vaccine to protect against HPAI H5N1. The vaccine was safe and well tolerated but was immunogenic only when administered with the adjuvant MF59. Putative protective antibody levels were not detectable in study participants 16 mo after receiving two doses [13], but significant boosting of antibody responses was seen upon administration of a third dose of vaccine. Although the geometric mean titers were low in all participants, higher seroconversion rates and cross-reactive antibodies were seen among participants who received the adjuvanted vaccine [14]. These studies demonstrated that an inactivated virus vaccine against H5N1 influenza viruses generated from antigenically related, low- pathogenicity avian viruses is weakly immunogenic but it can induce broadly reactive antibodies when administered in multiple doses with adjuvant [7,13,14].

A baculovirus-expressed recombinant H5 protein vaccine was evaluated in Phase 1 clinical studies in humans [8]. The vaccine was well tolerated but was poorly immunogenic; only 52% of participants who received two doses of 90 μg of vaccine developed a neutralizing antibody response to the vaccine.

Two adenoviral vectors expressing full-length H5 HA were tested in mice and poultry and, after two doses, were found to be immunogenic and protective against challenge with homologous and heterologous H5N1 *wt* influenza viruses [9,10]. These vaccine candidates elicited both humoral and cellular immune responses, and the protection against heterologous challenge was presumed to be mediated by the cellular immune response.

Multiple basic amino acid residues adjacent to the HA1-HA2 proteolytic cleavage site of HA of HPAI H5 and H7 viruses constitute a virulence motif that makes the HA cleavable by intracellular and extracellular proteases, thereby altering virus tropism and its ability to spread systemically (reviewed in [48]). Several investigators have developed experimental H5N1 influenza vaccines in which the HA cleavage site sequence is modified, resulting in the generation of a low-pathogenicity seed virus that can be handled under standard BSL-2 laboratory containment used for the production of current interpandemic human influenza vaccines [11,12]. Li and colleagues [15] reported the generation of two live, attenuated, *ca,* temperature-sensitive *(ts)* vaccine viruses based on two 1997 human H5N1 influenza isolates. The vaccine viruses were immunogenic in ferrets and retained the antigenicity of the *wt* parental viruses, demonstrating that production of live, attenuated recombinant H5 viruses containing a modified HA and the A/Ann Arbor/6/60 *ca* (AA *ca*) internal protein genes is a useful approach to the generation of vaccines against HPAI viruses. However, these vaccines were not tested in mice or humans. Subbarao and colleagues [11] reported the use of the same general approach to produce an inactivated experimental H5N1 influenza vaccine based on a 1997 H5N1 virus. The seed virus was generated by reverse genetics in 293T cells, a substrate that is not acceptable for use in human vaccines. Subsequently, Webby and colleagues [12] generated a recombinant H5N1 influenza virus in Vero cells, in which the modified HA and NA genes were derived from A/Hong Kong/213/2003 (H5N1) and the internal protein genes were derived from A/Puerto Rico/8/34 (H1N1). This vaccine was immunogenic in mice when administered with incomplete Freund's adjuvant and provided a dose-dependent protection against heterologous *wt* H5N1 viruses [16]. A similarly generated inactivated subvirion vaccine, based on A/Vietnam/1203/2004 (H5N1) has been evaluated in Phase 1 studies in healthy adult volunteers [17]. Although a correlation was observed between the dose of vaccine and immunogenicity, the vaccine was poorly immunogenic and only 54% to 58% of individuals immunized with two 90 μg doses of the vaccine developed antibody responses that had been predicted to be protective in two assays. The vaccine is now being evaluated in children and in the elderly. Evaluation of strategies to improve immunogenicity of the inactivated subvirion vaccine with adjuvants and dose-sparing regimens such as intradermal delivery, are ongoing. Thus, to date, the immunogenicity of inactivated virus vaccines that were evaluated in clinical trials is suboptimal and require at least two doses and/or the use of adjuvants.

We have been developing live, attenuated influenza virus vaccines because they have properties that make them attractive vaccines for the prevention of pandemic influenza in humans, including (1) stimulation of an immune response in influenza A virus-naïve individuals following a single dose of vaccine; (2) safety for fully susceptible seronegative individuals; (3) induction of cross-reactive immune responses; (4) poor transmissibility; and (5) genetic and phenotypic stability [18,19,20,21]. In the present study, three candidate live, attenuated H5N1 vaccine viruses were generated following rescue in Vero cells. Each of these vaccines, produced in eggs, contains a different H5 HA (from 1997, 2003, and 2004 H5N1 viruses) with the multibasic motif deleted, an accompanying avian N1 NA, and the set of six internal protein genes of the master donor virus AA *ca*. The three H5N1 *ca* viruses bear the phenotypes predicted by the modification engineered in the HA gene (trypsin dependence) and those specified by the internal protein genes of the AA *ca* virus (*ts* and attenuation *[att]*). The candidate H5N1 *ca* vaccine viruses were evaluated for immunogenicity in mice and for efficacy in mice and ferrets. The breadth of immunity that could be induced by immunization with live, attenuated H5N1 vaccines was determined in cross-protection studies using genetically and antigenically diverse H5N1 viruses isolated over an eight-year period representing different clades for challenge.

## Methods

### Viruses

The H5 HA and N1 NA of the reassortant H5N1 2003 and 2004 *ca* vaccine viruses were derived from influenza A/HK/213/2003 (H5N1 2003 *wt*) and A/VN/1203/2004 (H5N1 2004 *wt*) viruses, respectively, and the internal protein genes came from the AA *ca* donor virus. The H5N1 1997 reassortant vaccine candidate's H5 HA was derived from A/HK/491/1997 (H5N1 1997 *wt*), its N1 NA from A/HK/486/1997 (H5N1), and the remaining gene segments from AA *ca*. A/Beijing/262/95 *ca* (H1N1), which was generated by MedImmune Vaccines for seasonal influenza, was used as a control in evaluating the neurotropism of the viruses. A/New Caledonia/99 *ca* (H1N1) was generated by MedImmune Vaccines and was used as a control for evaluating vaccine efficacy in ferrets. A/Ann Arbor/6/60 (H2N2) *wt* (AA *wt*) and AA *ca* viruses were obtained from MedImmune Vaccines. Additional influenza A viruses used for challenge studies were A/Vietnam/JPHN30321/2005 (H5N1), which belongs to clade 1 [4], and A/Indonesia/05/2005 (H5N1), which belongs to clade 2 (R. Donis, Influenza Branch, Centers for Disease Control and Prevention, Atlanta, Georgia, United States, personal communication). The *wt* H5N1 viruses used in this study were kindly provided by N. Cox and A. Klimov, Influenza Branch, Centers for Disease Control and Prevention.

Virus stocks for the *wt* viruses were propagated in the allantoic cavity of 9- to 11-day-old embryonated specific pathogen-free (SPF) hen's eggs at 37 °C. The allantoic fluids from eggs inoculated with *wt* viruses were harvested 24 h postinoculation and tested for hemagglutinating activity. Eggs inoculated with *ca* reassortant viruses were incubated at 33 °C and were harvested 3 d postinoculation. Infectious allantoic fluids were pooled, divided into aliquots, and stored at −80 °C until use. The 50% tissue culture infectious dose (TCID_50_) for each virus was determined by serial titration of virus in Madin-Darby canine kidney (MDCK) cells and calculated by the method developed by Reed and Muench [22].

All experiments, including animal studies with infectious *wt* H5 avian influenza viruses and the reassortant viruses, were conducted using enhanced BSL-3 containment procedures in laboratories approved for use by the USDA and Centers for Disease Control and Prevention. Animal experiments were approved by the National Institutes of Health Animal Care and Use Committee. Experimental studies in chickens were approved by the USDA (SEPRL) Animal Care and Use Committee.

### Plasmids and Transfections

cDNAs generated by RT-PCR from all six internal protein gene segments of the AA *ca* were cloned into the plasmid pAD3000. This plasmid is a derivative of plasmid pHW2000 [23] and contains pol I and pol II promoters for the expression of vRNA and mRNA, respectively, from the full-length viral gene segment insert. Similarly, cDNAs derived by RT-PCR of the *wt* H5 genes, with a deletion of the sequence encoding four basic amino acids at the HA1–HA2 cleavage site (Table 1) and the *wt* N1 genes from the various H5N1 viruses were cloned into pAD3000. Sequences of all of the inserts were confirmed. For each H5N1 reassortant virus, the six plasmid DNAs encoding the internal protein genes of the AA *ca* were combined with the two plasmids encoding the modified H5 gene and *wt* N1 gene, and Vero cells were transfected with the eight-plasmid mixture by electroporation. Seed virus stocks were generated by three rounds of cloning by limiting dilution in SPF eggs, followed by expansion in SPF eggs. Viruses that derive gene segments from different parent viruses are referred to as reassortant viruses whether they were generated by reverse genetics or genetic reassortment.

**Table 1:**
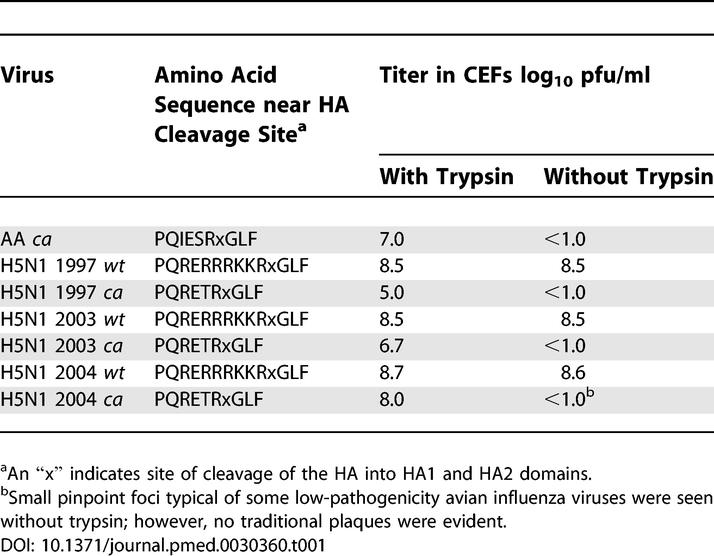
H5N1 *ca* Viruses without the Multibasic Amino Acid Motif in the HA Require Trypsin for Plaque Formation in CEFs

### Phenotypic Analysis of the Reassortant Viruses

The *ts* phenotype of the parent and reassortant viruses was assessed by evaluating viral replication or cytopathic effects (CPEs) in primary chick kidney cells at 33 °C and 39 °C. Viruses that displayed 100-fold or greater reduction in viral titers at high temperatures (≥ 39 °C) compared with that observed at the permissive temperature (33 °C) were considered *ts*.

The *ca* phenotype of the parent and reassortant viruses were determined by comparing their infectivity in primary chick kidney cells at 25 °C and 33 °C. Cold-adapted viruses can replicate efficiently at low temperatures; the *ca* phenotype is defined as a less than 100-fold reduction of virus titer at 25 °C compared with that at 33 °C. Cells incubated at 33 °C or 39 °C were examined for CPE 6 d postinfection and cells incubated at 25 °C were examined for CPE 10 d postinfection. The level of replication of the H5N1 *wt* and AA *ca* reassortant viruses were within 10- to 100-fold of each other at 25 °C and 33 °C, indicating that the *ca* phenotype was not a discriminating phenotype among these viruses; it has been observed that several *wt* human influenza viruses are able to replicate efficiently at 25 °C (unpublished data). It was therefore not surprising that this observation was made with the *wt* H5N1 viruses. This is a reflection of the variability of the biology of influenza viruses in nature.

### Plaque Assay with and without Trypsin

Chick embryo fibroblast (CEF) cells in 6-well tissue culture plates were inoculated with 0.1 ml of virus serially diluted in Leibovitz (L-15) medium. Virus was adsorbed to cells for 1 h, with shaking every 15 min. Wells were overlaid with 1.8% w/v Bacto agar (Difco, BD Diagnostic Systems, Palo Alto, California, United States) mixed 1:1 with 2× Medium 199 containing antibiotics and fungizone, with or without 0.6 μg/ml trypsin (Sigma, St. Louis, Missouri, United States). Plates were inverted and incubated for 2–3 d. Wells were then overlaid with 1.8% w/v Bacto agar mixed 1:1 with 2× Medium 199 containing 0.05 mg/ml neutral red, and plates were incubated for two additional days to visualize plaques.

### Pathogenicity and Infectivity Studies in Chickens

To determine high or low-pathogenicity of the H5N1 *ca* viruses, the standard intravenous lethality test was used [24]. Briefly, the H5N1 *wt* (1997 *wt,* 2003 *wt,* and 2004 *wt*) and reassortant H5N1 *ca* viruses were administered intravenously to groups of eight 4-wk-old White Plymouth Rock chickens at a standard dose of 0.2 ml of a 1:10 dilution of stock virus and were monitored for mortality up to ten days postinfection (p.i.) [11]. To determine infectivity via a simulated natural route of exposure, a separate group of chickens were inoculated intranasally with 10^6^ TCID_50_ of H5N1 *wt* and reassortant H5N1 *ca* viruses. Oropharyngeal and cloacal swabs were collected for virus isolation on day 3 p.i. or on the day of death in chickens that died before day 3. On day 14 p.i., all surviving chickens were euthanized and bled, and sera were tested for evidence of seroconversion by agar gel precipitin assays.

### Pathogenicity Studies in Mice

To determine the 50% lethal dose (LD_50_) of the different H5N1 *wt* and reassortant *ca* viruses, groups of 4- to 6-wk-old female BALB/c mice were anesthetized and infected intranasally with serial 10-fold dilutions of the viruses in 50 μl. Mice were monitored daily for 14 days p.i. for mortality.

To assess the ability of the viruses to replicate in different organs, groups of 24 female BALB/c mice were inoculated intranasally with 10^6^ TCID_50_ of H5N1 2004 *wt,* H5N1 2004 *ca,* or A/Beijing/95 *ca* (H1N1) viruses. Four mice from each group were euthanized on days 2, 4, 6, 8, 10, and 12 p.i. However, organs were collected only on days 2 and 4 from mice that had received the H5N1 2004 *wt* virus, since none of these mice survived beyond day 4. Lungs, nasal turbinates, and brains were harvested, weighed, and homogenized in L-15 medium containing antibiotics to make a 10% w/v tissue homogenate. Tissue homogenates clarified by low-speed centrifugation were titered in 24- and 96-well culture plates containing MDCK cells; titers are expressed as log_10_ TCID_50_/g of tissue.

### Replication in Ferrets

The ability of H5N1 *ca* and *wt* viruses to replicate in ferrets were compared. Groups of three 10- to 12-wk-old ferrets (Harlan, Madison, Wisconsin, United States) were pre-bled and tested by hemagglutination assay and were found to be negative for antibodies to H3N2 influenza. Each ferret was inoculated intranasally with 10^7^ TCID_50_ of H5N1 *ca* vaccine viruses or H5N1 *wt* viruses or AA *ca* in a volume of 1 ml (0.5 ml per nostril). Ferrets were monitored daily for clinical signs of influenza infection, and body temperatures were recorded twice daily. On day 3 postinoculation, ferrets were euthanized and nasal turbinates, lung, and brain tissue were harvested. The tissues were homogenized, serial 10-fold dilutions were prepared, and 0.1 ml of each dilution was inoculated into four 9- to 11-d-old embryonated SPF hen's eggs. Eggs were incubated at 33 °C for 72 h for the *ca* vaccine virus or 37 °C for 24 h for *wt* virus. Allantoic fluid from each egg was subjected to hemagglutination assay using 0.5% turkey erythrocytes. Virus titers were determined as 50% egg infectious doses (EID_50_) per gram of tissue using the Reed-Muench method.

### Evaluation of Immunogenicity of the Vaccine Candidates in Mice

Groups of 4- to 6-wk-old female BALB/c mice were immunized with 50 μl containing 10^6^ TCID_50_ of each reassortant virus, and sera were collected from these mice 4 wk later. Some groups of mice received two doses of 50 μl containing 10^6^ TCID_50_ of H5N1 2004 *ca* 28 d apart, and sera were collected 4 wk after the second dose. Serum samples were tested for the titer of HAI antibodies by standard methods using 4 HA units of *wt* virus in V-bottom 96-well microtiter plates with 0.5% turkey erythrocytes or with a 1% suspension of horse erythrocytes [25]. Neutralizing antibody titers were also evaluated in these samples by a microneutralization assay. Serial 2-fold dilutions of heat-inactivated serum were prepared, beginning with a 1:10 dilution. Equal volumes of serum and virus were mixed and incubated for 60 min at room temperature. The residual infectivity of the virus-serum mixture was determined in four wells per dilution. Neutralizing titer was defined as the reciprocal of the highest dilution of serum that completely neutralized infectivity of 100 TCID_50_ of the appropriate *wt* H5N1 virus for MDCK cells. Infectivity was identified by the presence of CPE on day 4.

### Evaluation of the Efficacy of the Vaccine Candidates

#### Protection from lethality in mice.

Groups of eight mice that received either a single dose of 10^6^ TCID_50_ of the H5N1 *ca* vaccine candidate or L-15 medium (mock-immunized) were challenged with 50, 500, and 5,000 LD_50_ of either the H5N1 1997 *wt* or the H5N1 2004 *wt* virus. The H5N1 2003 *wt* virus was not used in these studies because it was not lethal for mice. In addition, mice that received a single dose of H5N1 2004 *ca* virus were also challenged with two additional, more current antigenic variant H5N1 viruses that were isolated in 2005, A/VN/JPHN30321/2005 (H5N1) and A/Indonesia/05/2005 (H5N1). The mice were monitored daily for 21 d p.i.

#### Protection from replication of the challenge virus in the respiratory tract in mice.

The level of pulmonary viral replication was also evaluated and compared in separate groups of mice that received one or two doses of the H5N1 2003 *wt* or H5N1 2004 *ca* vaccine viruses. Mice that had received a single dose of 10^6^ TCID_50_ of the immunizing virus were challenged with 10^5^ TCID_50_ of H5N1 1997 *wt,* H5N1 2003 *wt,* or H5N1 2004 *wt* viruses on day 28 p.i. Lungs were harvested 2 d later, homogenized, and titrated on MDCK cells as previously described. Mice that received two doses of the immunizing virus four weeks apart were challenged with the H5N1 *wt* viruses on day 56 p.i., their lungs were harvested 2 days later and were homogenized and titered on MDCK cells. Log-transformed viral titers were compared using the Mann-Whitney U test.

In a separate experiment, mice that received two doses of 10^6^ TCID_50_ of H5N1 1997 *ca,* H5N1 2003 *ca,* or H5N1 2004 *ca* viruses 4 wk apart were challenged on day 56 p.i. with 10^5^ TCID_50_ of homologous and heterologous *wt* H5N1 viruses, including A/Indonesia/05/2005 (H5N1). The lungs and brains were harvested from these mice 4 d later and were homogenized and titered on MDCK cells.

#### Protection from replication of challenge viruses in ferrets.

Groups of nine 6-wk old ferrets were inoculated intranasally with 10^7^ TCID_50_ of either H5N1 1997 *ca,* H5N1 2004 *ca,* or L-15 medium (mock-immunized), in a volume of 0.5 ml/nostril, on days 0 and 28. In order to determine whether the internal protein genes of the AA *ca* virus contributed to protection, an additional group of ferrets immunized with A/New Caledonia/99 *ca* (H1N1) was included. Serum samples were collected from ferrets on day 18 after the second vaccination, and HAI antibody titers against the relevant challenge virus were determined using horse erythrocytes. At 1 mo after the second immunization, ferrets were challenged intranasally with 10^7^ TCID_50_ of either H5N1 1997 *wt,* H5N1 2004 *wt,* or A/Indonesia/05/2005 (H5N1). Ferrets that had been immunized with A/New Caledonia/99 *ca* (H1N1) were challenged only with H5N1 1997 *wt*. At 3 d postchallenge, ferrets were euthanized, and brain, lungs, and nasal turbinates were harvested and homogenized. Tissue homogenates were titered on MDCK cells.

Log-transformed viral titers were compared using the two-tailed Student's t-test. The correlation of HAI titer with viral titer in the various tissues was determined by calculation of the Spearman rank correlation coefficient.

## Results

### Generation of H5N1 *ca* Viruses

Reassortant viruses with a modified H5 HA gene and N1 NA gene derived from H5N1 1997, 2003, and 2004 viruses and remaining gene segments from AA *ca* were generated by reverse genetics [23]. The HA gene was modified by replacing the multibasic amino acid motif with the sequence seen in avian influenza viruses that are not highly pathogenic in chickens (Table 1) [11]. The nucleotide sequence of each gene segment of the three viruses was identical to the sequence of the corresponding gene insert in the parent viruses, with the exception of a single coding change each in the PA gene segment of the 2004 *ca* and 1997 *ca* viruses. The five mutations in the AA *ca* virus that specify *ts* and *att* phenotypes were present in the H5N1 *ca* viruses [26,27]. The H5N1 *ca* vaccine viruses were antigenically similar to their respective *wt* parent viruses by HAI test with postinfection ferret antisera (unpublished data). The phenotypic characteristics of the H5N1 *ca* viruses were also evaluated in vitro and in vivo in chickens, mice, and ferrets.

### In Vitro Phenotypes: Trypsin Dependence and Temperature Sensitivity

The HA gene in the H5N1 *ca* viruses differed from the HA gene in the corresponding *wt* parent viruses by the absence of the multibasic amino acid cleavage site. The presence of this motif in an influenza A virus of the H5 or H7 subtype confers the ability to plaque efficiently in CEF cells in the absence of trypsin [28–30]; the H5N1 1997 *wt,* H5N1 2003 *wt,* and H5N1 2004 *wt* viruses demonstrated this property (Table 1). The H5N1 *ca* viruses failed to plaque in CEF cells in the absence of trypsin, consistent with the absence of the multibasic cleavage site motif in the HA protein (Table 1) [29].

Extensive experience with H1N1 and H3N2 reassortant viruses and an H9N2 reassortant virus that derived the six internal protein gene segments from the AA *ca* virus has shown that the *ts* and *att* phenotypes are specified by these gene segments [19,26,27,31,32]. The H5N1 *ca* viruses and the AA *ca* parent virus were restricted in replication at 39 °C *(ts);* in contrast, the *wt* H5N1 parent viruses replicated equally well at 33 °C and 39 °C (Table 2). Thus, the three H5N1 *ca* viruses demonstrated the *ts* phenotype.

**Table 2:**
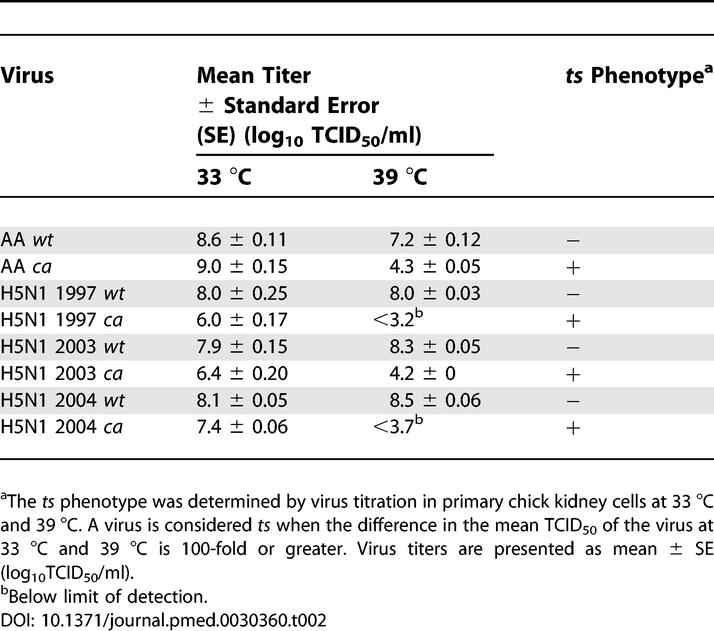
Evaluation of the *ts* Phenotype of Wild-Type and Reassortant H5N1 *ca* Influenza Viruses

### Attenuation In Vivo in Chickens, Mice, and Ferrets

#### Level of attenuation in chickens following intravenous administration.

The H5N1 1997 *wt,* H5N1 2003 *wt,* and H5N1 2004 *wt* viruses were highly pathogenic for chickens, findings that are consistent with the presence of multibasic amino acid motif in the HA [28–30] (Table S1). The H5N1 *ca* viruses were no longer lethal for chickens in the standard intravenous pathogenicity test, consistent with the absence of the multibasic cleavage site motif in the HA protein (Table S1) [29].

#### Level of attenuation in chickens following intranasal administration.

In order to determine whether H5N1 *ca* viruses would infect and cause disease in chickens exposed to the virus through a more natural route, the H5N1 *wt* and *ca* viruses were administered to chickens intranasally. The H5N1 *wt* viruses were recovered from cloacal and oropharyngeal swabs on day 3 following virus administration, and the infection was lethal for 8 of 8 chickens (Table S1). The H5N1 *ca* viruses were not lethal for chickens and did not appear to infect the chickens following intranasal administration, as indicated by the absence of detectable infectious virus in cloacal or oropharyngeal swabs and the absence of an antibody response to the virus (Table S1). The poor infectivity of the H5N1 *ca* viruses in chickens is likely the result of an inability of these *ts* viruses to replicate at the high body temperature of chickens (40–41 °C) and suggests that their use would not pose a threat to the poultry industry.

#### Lethality in mice.

When administered intranasally, the H5N1 1997 *wt* and H5N1 2004 *wt* viruses were highly lethal for mice (LD_50_ values of 10^2^ and 10^0.4^ TCID_50_, respectively), whereas the H5N1 2003 *wt* virus was lethal for a few mice at a very high dose (LD_50_ ≥ 10^6^ TCID_50_). Lethality for mice was completely abrogated in the three H5N1 *ca* viruses; none of the mice died even at the highest dose of virus administered (LD_50_ > 10^7^ TCID_50_).

#### Level of replication in the respiratory tract of mice.

The level of replication of the H5N1 1997 *ca* and H5N1 2004 *ca* viruses was lower than that of the corresponding H5N1 *wt* viruses in the upper and lower respiratory tract of mice (Table 3), and the differences in titer were similar to or exceeded the difference between the titers achieved in mice infected with the AA *wt* and *ca* viruses. The level of replication of the H5N1 2003 *wt* and *ca* virus did not differ significantly in the respiratory tract (Table 3 and unpublished data for days 2 and 4). The reasons for this observation are not clear, but it may be the result of a partial attenuation of the H5N1 2003 *wt* virus, which does not replicate as efficiently in the respiratory tract of mice as the H5N1 1997 *wt* and H5N1 2004 *wt* viruses, and may also partly explain why the H5N1 2003 *wt* virus was not lethal for mice in some studies [33], although other investigators have found the H5N1 2003 *wt* virus to be lethal in mice [16].

**Table 3:**
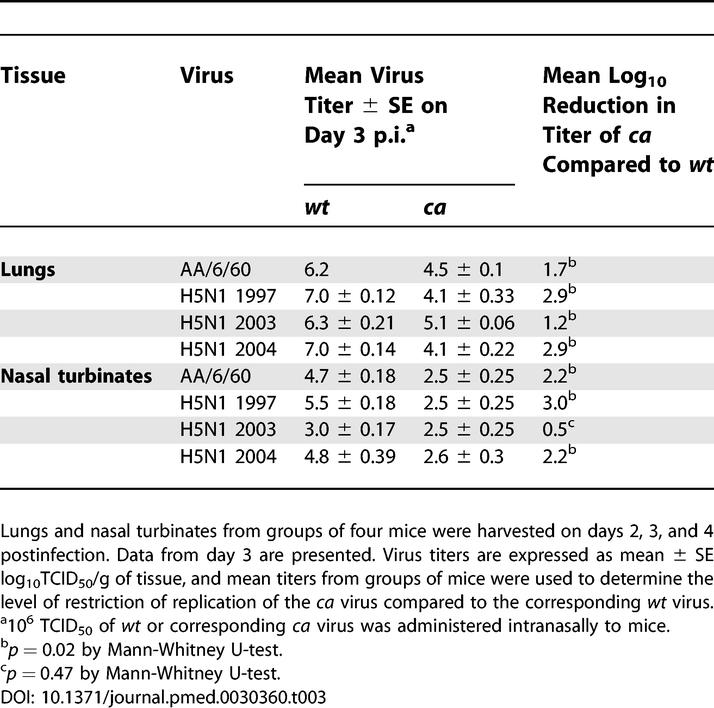
The H5N1 *ca* Viruses Are Restricted in Replication in the Respiratory Tract of Mice

#### Level of replication of H5N1 2004 *ca* virus in the brains of mice following intranasal administration.

**Figure 1:**
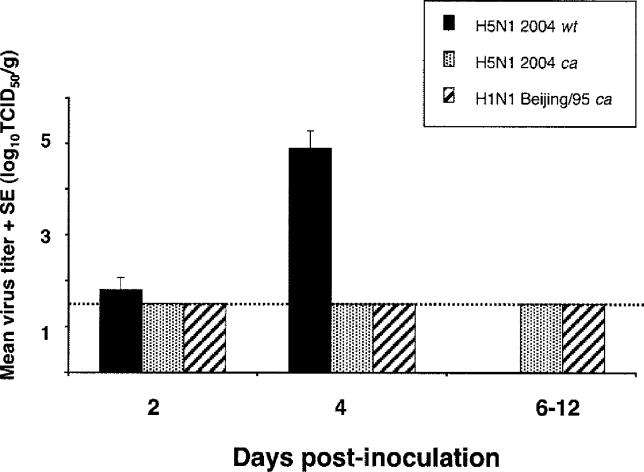
Replication of H5N1 2004 *ca* and 2004 *wt* Viruses in the Brains of Mice The H5N1 2004 *ca* virus, unlike the H5N1 2004 *wt* parent virus, is undetectable in the brain. Influenza A/Beijing/95 *ca,* an H1N1 *ca* reassortant virus, was also undetectable in the brains of mice. The lower limit of detection is indicated by the dashed horizontal line. Four mice per group were euthanized at each time point (days 2, 4, 6, 8, 10, and 12). Data from days 6 through 12 are combined in the figure because virus was not detected in any of the brain tissues harvested on these days.

Previous studies have established that some of the H5N1 *wt* viruses can be detected in the brains of mice following intranasal administration [33–38]. In order to determine whether the modifications in the HA gene and the presence of the internal protein gene segments from the AA *ca* virus altered the neurotropism of the H5N1 2004 *ca* reassortant viruses, we administered the H5N1 2004 *ca* virus, the H5N1 2004 *wt* virus, and an H1N1 *ca* virus (as a control) to mice by the intranasal route and determined the ability of these viruses to replicate in the respiratory tract or brain. A single dose of 10^6^ TCID_50_ of each virus was administered intranasally, and eight mice from each group were sacrificed on alternate days from day 2 to day 12 p.i. (Figure 1). The H5N1 2004 *wt* virus was detected in the brains of mice on days 2 and 4 p.i. with mean titers of 10^1.8^ and 10^4.9^ TCID_50_/g, respectively, but the H5N1 2004 *ca* virus and the *ca* virus were not. It should be noted that although the H5N1 2004 *ca* virus and the H1N1 *ca* virus were not recovered from the brain, they were isolated from the lungs and nasal turbinates (unpublished data), indicating that the H5N1 *ca* and H1N1 *ca* viruses replicated in the respiratory tract, but did not spread to the central nervous system of mice. Further studies are warranted to delineate the relative contribution(s) of the modified HA and the internal genes of AA *ca* to the observed lack of neurotropism of the H5N1 2004 *ca* virus. A similar lack of neurotropism of the H5N1 1997 *ca* virus was also observed (unpublished data).

#### Level of replication of H5N1 2004 *ca* and H5N1 1997 *ca* in ferrets.

The levels of replication of the H5N1 2004 *ca* and H5N1 1997 *ca* viruses compared to their respective *wt* parental H5N1 viruses in ferrets were determined. The H5N1 2004 *ca* and H5N1 2004 *wt* viruses replicated in the upper respiratory tract of ferrets but the H5N1 2004 *ca* virus was not detected in the lower respiratory tract or brain of any of the ferrets. In contrast, the H5N1 2004 *wt* virus replicated in the lungs and brains of ferrets (Table 4). The H5N1 1997 *wt* virus replicated efficiently in the upper respiratory tract of ferrets, and the H5N1 1997 *ca* virus was detected in the nasal turbinates of two out of three ferrets. The H5N1 1997 *ca* virus was not detected in the lower respiratory tract or brain of ferrets, unlike the H5N1 1997 *wt* virus, that was isolated from the lungs and brains of all three ferrets (Table 4). The H5N1 1997 *ca* and H5N1 2004 *ca* viruses were highly restricted in replication in the upper respiratory tract of ferrets compared to the corresponding *wt* viruses. The attenuation of the H5N1 2004 *ca* and H5N1 1997 *ca* vaccine viruses in the respiratory tract of ferrets was consistent with the observations in mice.

**Table 4:**
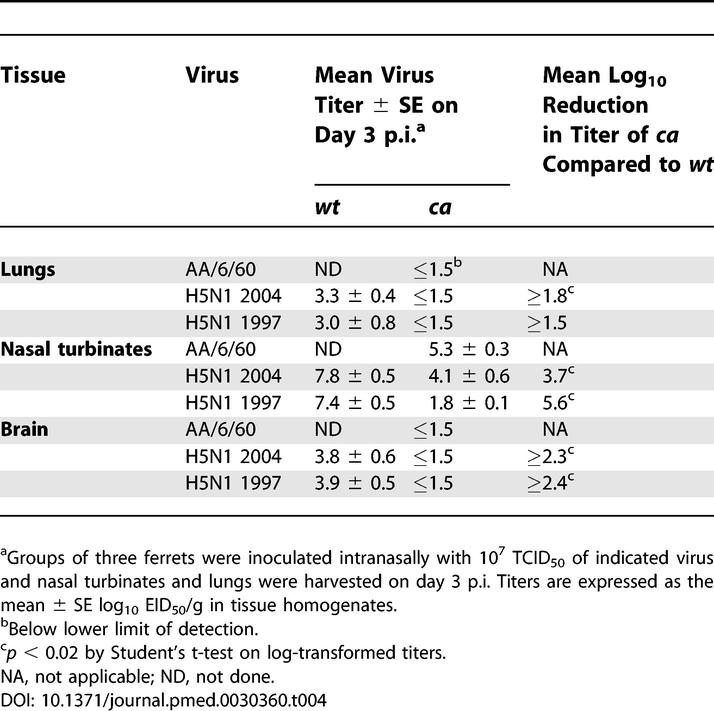
The H5N1 *ca* Viruses Are Attenuated in Ferrets

Thus, the H5N1 *ca* viruses demonstrated the properties predicted by the genetic modifications of removing the multibasic amino acid motif in the HA and the introduction of the internal gene segments from the AA *ca* virus. Studies were next undertaken to determine the immunogenicity and efficacy of these viruses as candidate vaccines.

### Immunogenicity of the H5N1 *ca* Virus Vaccine Candidates

Each of the H5N1 *ca* candidate vaccines was administered intranasally to mice at a dose of 10^6^ TCID_50_, and serum antibody responses at 28 d postimmunization were measured by HAI and microneutralization assays. The nonlethal H5N1 2003 *wt* virus was administered to a group of mice as a positive control. A single dose of vaccine was poorly immunogenic in mice, with low antibody responses in both assays (Table 5). However, ELISA antibodies were detected in the serum against recombinant H5 HA (unpublished data). The titer and cross-reactivity of the neutralizing and HAI antibody response was greater 4 wk after a second dose of vaccine administered 28 d after the first dose (Table 6), although replication of the second dose of vaccine was not detected in the respiratory tract (unpublished data). The H5N1 2003 *ca* and *wt* viruses elicited higher antibody titers against the homologous virus than the H5N1 1997 *ca* and H5N1 2004 *ca* viruses; these findings are consistent with previous observations that identified the amino acid asparagine at position 223 in the HA as a determinant of enhanced immunogenicity of the H5N1 2003 viruses [39].

**Table 5:**
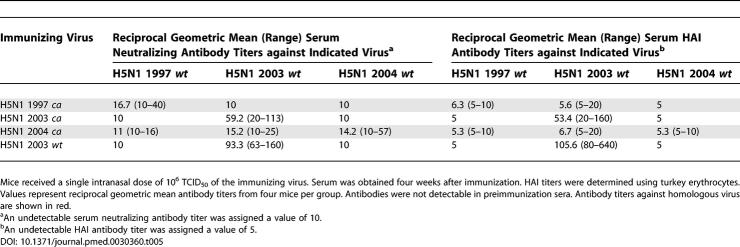
Serum Neutralizing and HAI Antibodies Elicited in Mice Following a Single Intranasal Dose of H5N1 *ca* Vaccine

**Table 6:**
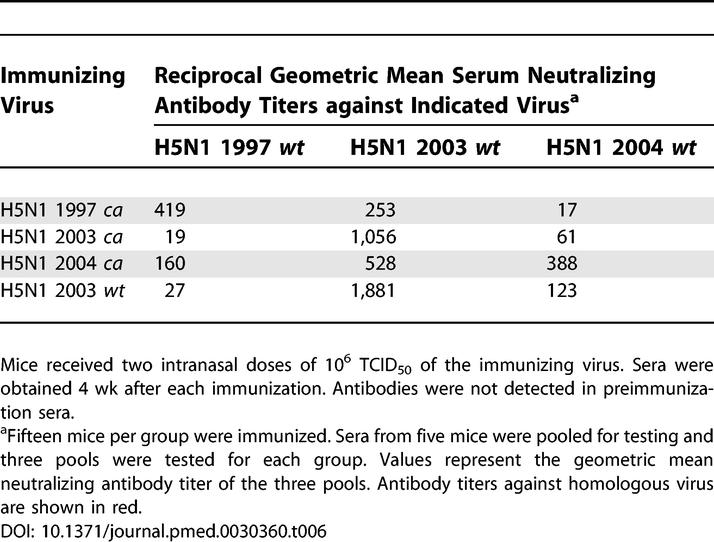
Serum Neutralizing Antibodies Elicited in Mice Following Two Doses of H5N1 *ca* Vaccine

An additional study was performed to determine the immunogenicity of one versus two intranasal doses of vaccine. In this study, one group of eight mice received a dose of vaccine on day 0, a second group received vaccine on days 0 and 28, and a third group was mock-immunized on day 0 and received vaccine on day 28. Serum was collected from all of the mice on days 27 and 54. The HAI antibody response 4 wk after a single dose of the H5N1 2004 *ca* vaccine was low. Responses to a single dose of vaccine continued to rise over 2 mo, however, and titers achieved 4 wk after a second dose of vaccine were higher than in mice that received a single dose of vaccine 8 wk earlier (Table 7). The pattern of HAI responses was the same whether the assay was performed with turkey or horse erythrocytes, but the HAI assay was more sensitive when performed with horse erythrocytes (Table 7).

**Table 7:**
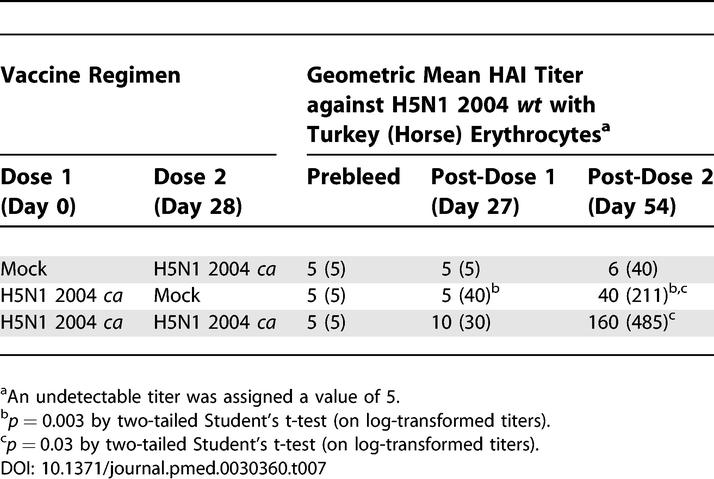
Geometric Mean HAI Antibody Titers in Mice Immunized with One or Two Doses of the H5N1 2004 *ca* Influenza Virus

### Efficacy of the H5N1 *ca* Virus Vaccine Candidates

#### Protection from lethality in mice.

**Figure 2:**
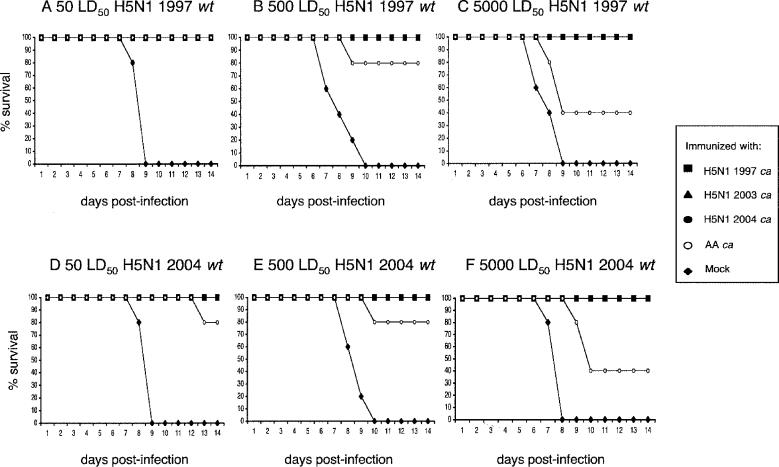
Survival of Mice Immunized with a Single Dose of H5N1 *ca* Vaccine Candidates Following Lethal Challenge with H5N1 1997 *wt* or H5N1 2004 *wt* Viruses Groups of eight mice that received a single dose of 10^6^ TCID_50_ of the H5N1 1997 *ca* (▪), H5N1 2003 *ca* (▵), H5N1 2004 *ca* (^), or AA *ca* (○) viruses, or were mock-immunized (L-15 medium; ♦) were challenged with the H5N1 1997 *wt* (A–C) or H5N1 2004 *wt* virus (D–F) at 50 (A and D), 500 (B and E), or 5,000 (C and F) LD_50_. The mice were monitored daily for 21 d p.i. Immunization with the H5N1 1997 *ca,* H5N1 2003 *ca,* and H5N1 2004 *ca* viruses resulted in 100% survival of the mice following challenge, so the symbols for these groups are superimposed.

All of the mice immunized with H5N1 *ca* vaccine viruses survived challenge with 50, 500, or 5,000 LD_50_ of homologous or heterologous H5N1 *wt* viruses, despite an undetectable neutralizing and HAI antibody response in mice 28 d following a single intranasal dose of 10^6^ TCID_50_ of the vaccines (Figure 2). Mock-immunized mice died between days 6 and 10 p.i. The AA *ca* virus that was the source of the six internal protein genes of the H5N1 *ca* vaccine viruses provided partial heterosubtypic protection from lethality following challenge with the H5N1 1997 and H5N1 2004 *wt* viruses. This protection decreased with increasing doses of challenge virus (Figure 2). The mechanism of protection from lethality that was observed in the absence of detectable serum neutralizing antibodies against H5N1 viruses is under investigation.

#### Protection from challenge virus replication in the respiratory tract of mice.

**Figure 3:**
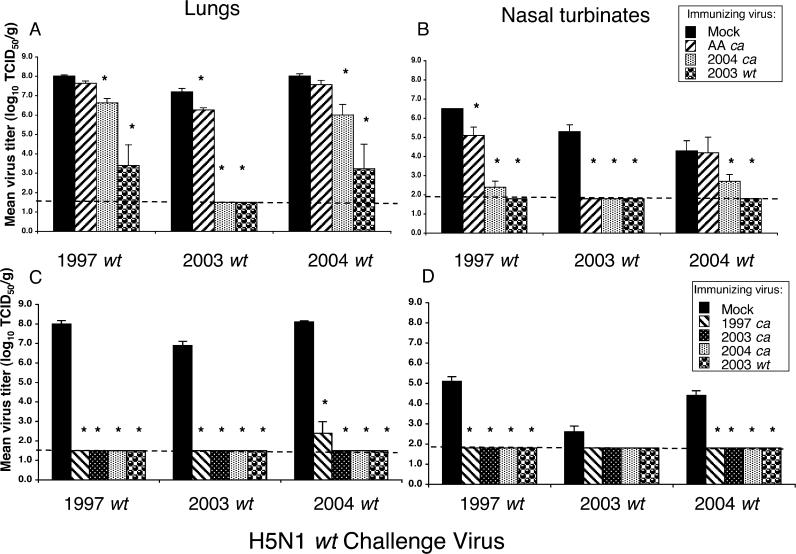
Replication of H5N1 *wt* Challenge Viruses in Mice Immunized with One or Two Doses of H5N1 *ca* Viruses (A and B) Mice that received a single dose of 10^6^ TCID_50_ of the immunizing virus were challenged with 10^5^ TCID_50_ of H5N1 1997 *wt,* H5N1 2003 *wt,* or H5N1 2004 *wt* viruses on day 28 p.i. Replication of challenge virus is shown in the lungs (A) and nasal turbinates (B). (C and D) Mice that received two doses of the immunizing virus 4 wk apart were challenged with the H5N1 *wt* viruses on day 56 p.i. Replication of challenge virus is shown in the lungs (C) and nasal turbinates (D). Lungs were harvested 2 d following challenge, homogenized, and titrated on MDCK cells. Groups of mock-immunized (L-15) mice and mice immunized with the nonlethal 2003 *wt* virus (single dose) or with AA *ca* were included as control groups. Four mice were evaluated in each group. The lower limit of detection is indicated by the dashed horizontal line. Log-transformed viral titers were compared using the Mann-Whitney U test. * *p* ≤ 0.05 compared to mock-immunized mice.

Although mice that received a single dose of vaccine were protected from lethality, they were not fully protected from replication of the H5N1 1997 *wt* or H5N1 2004 *wt* virus in the respiratory tract (Figure 3A and 3B). The titer of the challenge virus detected in the respiratory tract of mice immunized with the H5N1 2004 *ca* vaccine was significantly lower than in mock-immunized mice or mice immunized with the AA *ca* virus (Figure 3A and 3B), and the challenge virus did not spread to the brain (unpublished data). However, complete protection from pulmonary virus replication was not seen following a single dose of vaccine when mice were challenged with 10^6^ TCID_50_ or even 10^2^ TCID_50_ of H5N1 1997 *wt* or H5N1 2004 *wt* virus (unpublished data). Incomplete protection from pulmonary virus replication is consistent with the low neutralizing and HAI antibody response 28 d following a single dose of vaccine (Table 5). Interestingly, a single dose of the H5N1 2004 *ca* virus provided complete protection from replication of the H5N1 2003 *wt* virus in the respiratory tract (Figure 3A and 3B). In consonance with a higher level of neutralizing and HAI antibody response following two doses of vaccine (Tables 6 and 7), complete or near-complete protection from virus replication was observed in the respiratory tract of mice following two doses of vaccine (Figure 3C and 3D).

### Efficacy of the H5N1 *ca* Virus Vaccine Candidates against Antigenic Variant *wt* H5N1 Viruses Isolated in 2005

#### Efficacy in mice.

**Figure 4:**
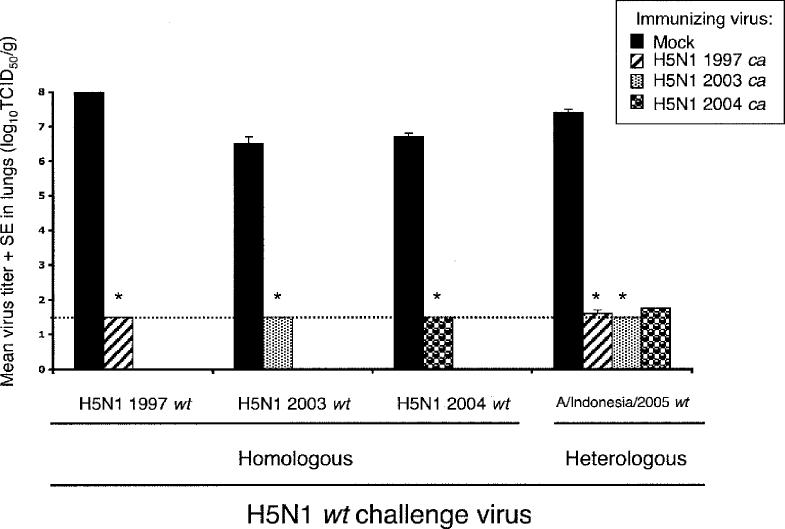
Immunization of Mice with Two Doses of H5N1 *ca* Viruses Provides Protection against Replication of Homologous and Heterologous H5N1 *wt* Viruses in the Lungs Mice that received two doses of 10^6^ TCID_50_ of H5N1 1997 *ca,* H5N1 2003 *ca,* or H5N1 2004 *ca* viruses 4 wk apart were challenged on day 56 p.i. with 10^5^ TCID_50_ of homologous and heterologous H5N1 *wt* viruses, including A/Indonesia/05/2005, a clade 2 H5N1 *wt* virus. The lungs were harvested from these mice 4 d later and were homogenized and titered on MDCK cells. Four mice were evaluated in each group. The lower limit of detection is indicated by the dashed horizontal line. * *p* < 0.02 compared to mock-immunized mice.

The H5N1 *ca* vaccines were also evaluated for their efficacy against challenge with newly emerged and antigenically distinct H5N1 strains, A/VN/JPHN30321/2005 (H5N1), designated a clade 1 virus, and A/Indonesia/5/2005 (H5N1), a clade 2 virus by the CDC [4]. A single intranasal dose of 10^5^ TCID_50_ of the H5N1 2004 *ca* vaccine provided complete protection from lethality following challenge (10^5^ TCID_50_) with A/VN/JPHN30321/2005 (H5N1) and A/Indonesia/5/2005 (H5N1) (unpublished data). Moreover, mice that received two doses (10^6^ TCID_50_/dose) of the H5N1 *ca* vaccines were protected from viral replication in the lungs and brains following challenge with 10^5^ TCID_50_ of A/Indonesia/5/2005 (H5N1) (Figure 4). This protection from viral replication was associated with high neutralizing antibody titers against A/Indonesia/5/2005 (H5N1) that were detected in the sera of all immunized mice that received two doses of the vaccines (geometric mean neutralizing antibody titers of 90, 174, and 104 for mice that were immunized with two doses of the H5N1 1997 *ca,* H5N1 2003 *ca,* and H5N1 2004 *ca,* respectively).

#### Efficacy in ferrets.

**Figure 5:**
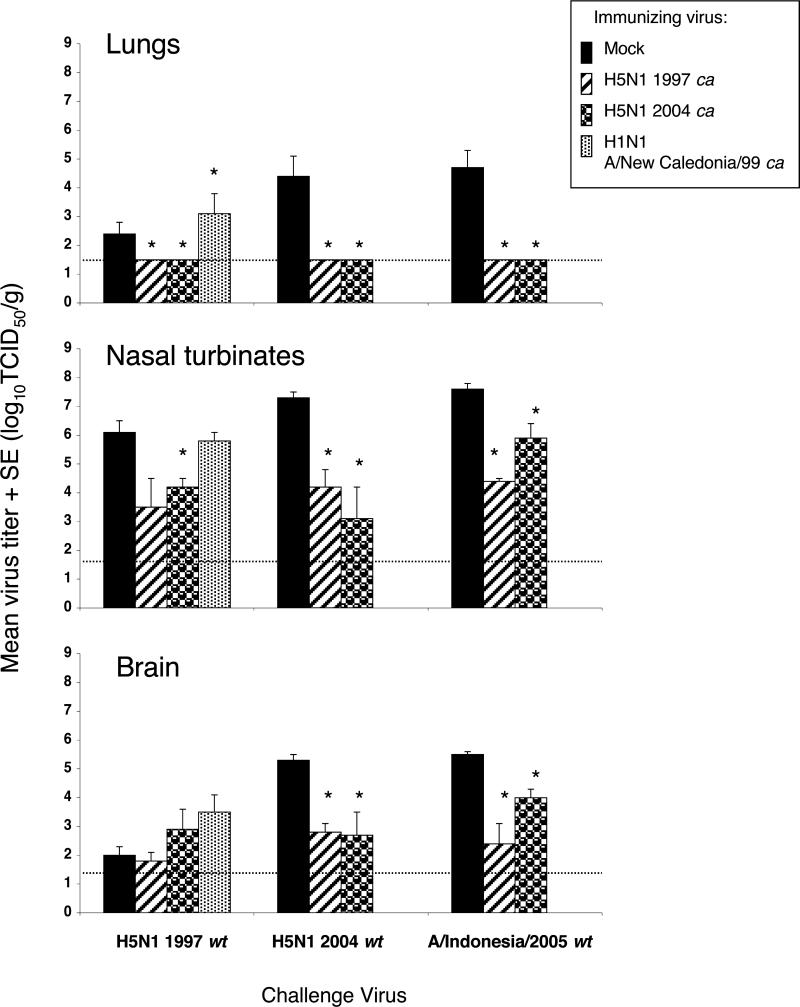
Efficacy in Ferrets of Two Doses of H5N1 *ca* Viruses against Challenge with Homologous and Heterologous H5N1 *wt* Viruses Ferrets were immunized with two doses of 10^7^ TCID_50_ of H5N1 1997 *ca* or H5N1 2004 *ca* viruses 1 mo apart and challenged 1 mo later with 10^7^ TCID_50_ of either H5N1 1997 *wt,* H5N1 2004 *wt,* or A/Indonesia/05/2005 (H5N1) *wt*. In order to determine the contribution of the internal protein genes of the AA *ca* virus to protection, an additional group of ferrets was immunized with H1N1 A/New Caledonia/99 *ca* and challenged with H5N1 1997 *wt*. These ferrets were not challenged with H5N1 2004 *wt* or A/Indonesia/05/2005 (H5N1) *wt*. Lungs, nasal turbinates, and brains were harvested 3 d after challenge and were homogenized and titered on MDCK cells. Three ferrets per group were evaluated. The lower limit of detection is indicated by the dashed line. *p < 0.05 compared to mock-immunized ferrets.

Ferrets that received two doses (10^7^ TCID_50_/dose) of the H5N1 *ca* vaccines were completely protected from viral replication in the lungs following homologous or heterologous challenge; the latter included challenge with 10^7^ TCID_50_ of A/Indonesia/5/2005 (H5N1) (Figure 5). Virus titers in the nasal turbinates of ferrets immunized with H5N1 2004 *ca* or H5N1 1997 *ca* were statistically significantly lower than those of the mock-immunized ferrets following challenge with H5N1 2004 *wt* or A/Indonesia/05/2005 (H5N1). Virus was detected in the brains of these ferrets at statistically significantly lower titers than in mock-immunized animals. A possible explanation for the presence of virus in the brains of these ferrets is that challenge inoculum was present in the olfactory bulb. Since different areas of the brain were not processed separately, this cannot be confirmed. There was a strong correlation between the titers of virus in these tissues and the immune response at day 18 after the second immunization (*p* < 0.000001 and *p* = 0.0035, respectively).

Thus, immunization with two doses of an H5N1 *ca* vaccine possessing the surface glycoprotein genes of the 1997 virus provided a high level of protection in mice and ferrets against H5N1 viruses that had evolved in nature over 8 y and that belonged to different phylogenetic clades.

## Discussion

Reverse genetics technology [23,40,41], used to generate influenza viruses from cells cotransfected with plasmids expressing influenza virus gene segments, has been applied to vaccine development since 1998 [11,12,15]. The technology is especially valuable for the generation of vaccines against HPAI viruses because it permits modification of gene(s) to remove specific virulence motifs such as the highly cleavable multibasic amino acid motif in the HA protein. The generation of a candidate vaccine for human use requires transfection of cells that are qualified for use in generation of vaccines for use in humans; subsequent amplification and biological cloning are carried out in SPF embryonated hen's eggs. The H5N1 viruses that were targeted for vaccine development were selected in consultation with public health authorities and represent viruses isolated in 1997, 2003, and 2004 from human cases of H5N1 infection. These *wt* viruses were antigenically distinguishable from each other using postinfection ferret antisera [4]. Live, attenuated vaccine candidates were generated against each of the three *wt* viruses using a common strategy. The HA gene of each of the live, attenuated vaccine candidates was modified to remove the multibasic virulence motif as described in earlier publications [11,12,15], and the six internal protein genes were derived from the highly attenuated AA *ca* donor virus, as described in [15]. The H5N1 *ca* candidate vaccine viruses displayed the *ts* and *att* phenotypes that are specified by the internal protein genes of the AA *ca* [26,27], and the viruses failed to plaque in CEF cells in the absence of trypsin, consistent with the absence of the multibasic cleavage site in the HA.

The safety of the H5N1 *ca* vaccine viruses was established in mice and ferrets. The H5N1 1997 *ca* and H5N1 2004 *ca* viruses were not lethal for mice, and when compared with the corresponding H5N1 *wt* viruses, they were restricted in replication in the upper and lower respiratory tract of mice. The acquisition of the six AA *ca* internal protein genes by the H5N1 1997 *wt* and H5N1 2004 *wt* viruses attenuated these viruses for mice. The level of restriction of replication of the H2N2 and H5N1 *wt* viruses specified by the six AA *ca* internal protein genes was comparable, indicating that the acquisition of these genes reproducibly attenuated the H5N1 avian influenza viruses for mice. This has also been seen for an H9N2 avian influenza virus [32]. Thus, the observation that the H5N1 2003 *wt* and *ca* viruses replicated to similar levels in mice was unexpected. Since the H5N1 2003 *wt* virus itself replicated to a low level and exhibited minimal lethality, it is reasonable to suggest that this H5N1 *wt* virus contains sequences that restrict its replication in mice. If these sequences occur in one or more of the internal protein genes of the virus, it is not surprising that substitution of these attenuating sequences with the six internal protein genes of the AA *ca* virus did not result in further attenuation. This suggestion will need experimental verification. The *att* phenotype was also seen in the ferret model: the H5N1 2004 *wt* and H5N1 1997 *wt* viruses replicated to high titers in the upper and lower respiratory tract, while the H5N1 2004 *ca* and H5N1 1997 *ca* viruses were attenuated in the nasal turbinates and were undetectable in the lungs and brain of ferrets. The similar levels of attenuation displayed by H5N1 2004 *ca* and H5N1 1997 *ca* in mice and ferrets indicate that either animal model could be used to demonstrate the attenuation of reassortant viruses bearing internal protein genes from the AA *ca* donor virus. Thus, the H5N1 *ca* viruses were safe for the respiratory tract of mice and ferrets, and additional studies indicated that the H5N1 2004 *ca* virus did not spread from the respiratory tract of mice or ferrets to the brain, while the H5N1 2004 *wt* virus readily infected the brains of these animals. The H5N1 *ca* viruses, in contrast to the H5N1 *wt* viruses, were not lethal for chickens in the standard intravenous pathogenicity test or when administered intranasally, indicating that these viruses should not pose a threat to agriculture. However, the H5N1 *ca* viruses would not be suitable candidate vaccines for use in poultry, because they fail to replicate and to induce an antibody response in chickens.

Despite a high level of replication in the respiratory tract of mice, the H5N1 *ca* candidate vaccine viruses were weakly immunogenic in mice following a single dose. Neutralizing antibody titers induced following a single dose continued to rise for 1–2 mo following immunization, indicating that the functional antibody response develops slowly in mice. A second dose of vaccine resulted in a significant boost in antibody titer and in cross-reactivity of the antibody with antigenically distinguishable H5N1 viruses. If similar antibody kinetics are observed in clinical trials, a single dose of vaccine may suffice to confer protection, but the protection may be slow to develop. A boost in antibody titers followed a second dose of vaccine despite the fact that replication of the second dose of vaccine was not detected. Thus, a delayed maturation of functional antibody occurred in response to the H5N1 *ca* vaccine viruses compared to that seen with other influenza A viruses [32,42]. The delayed antibody response had a correlate in efficacy studies.

A single dose of H5N1 *ca* vaccines provided complete protection from lethality following challenge with homologous and heterologous H5N1 *wt* viruses and prevented systemic spread of the *wt* challenge viruses to the brain. A single dose of H5N1 *ca* vaccines also resulted in statistically significant restriction of replication of the *wt* challenge viruses in the lungs that greatly exceeded that induced by infection with the heterosubtypic AA *ca* (H2N2) virus, thus indicating that this protection was HA- and NA-specific. However, a single dose of vaccine did not induce complete protection against replication of challenge virus in the respiratory tract. In a clinical setting, this partial protection in mice may translate to protection from severe illness and death in humans. Two doses of the H5N1 *ca* candidate vaccine viruses induced high titers of antibody and provided complete protection from pulmonary replication of homologous and heterologous H5N1 *wt* viruses. Replication of the second dose of vaccine virus could not be detected, but antigenic stimulation was sufficient to provide a boost in immune response. Two doses of the H5N1 *ca* vaccine in mice and in ferrets provided complete protection from pulmonary replication of homologous and heterologous H5N1 *wt* viruses, including A/Indonesia/05/2005 (H5N1). In mice, two doses of the H5N1 *ca* vaccine provided complete protection from systemic dissemination of challenge virus. Virus was detectable in the brains of ferrets following challenge with a high dose of H5N1 *wt* virus, but viral titers were statistically significantly reduced compared to mock-immunized animals. Similar to the observations in mice immunized with the AA *ca* virus, the protection in ferrets against pulmonary replication of challenge virus afforded by the H5N1 *ca* vaccines exceeded that observed when ferrets were immunized with an H1N1 *ca* virus bearing the same AA *ca* internal protein genes, which suggests that the protection is specific to the H5 HA.

It is not possible to predict the evolution of the H5 HA or to predict which strain, if any, will become pandemic. Therefore, an H5N1 vaccine should elicit a cross-protective immune response against a range of H5N1 viruses, including newly emerged strains. In addition, a pandemic vaccine needs to be preselected before the pandemic virus emerges in the population and characterized in humans for safety and immunogenicity, since it is highly unlikely that a vaccine can be generated, manufactured, and characterized rapidly enough to thwart spread of a pandemic strain. The assumption underlying this approach is that the preselected pandemic vaccine virus can prevent disease caused by antigenically and genetically divergent viruses belonging to the same subtypes that appear in nature. In the present study, we were able to directly evaluate this assumption, and our data on the H5N1 *ca* vaccines are very encouraging in this regard. A single dose of an H5N1 *ca* vaccine containing the 1997 H5 HA provided complete protection from lethality in mice following challenge with homologous and heterologous H5N1 *wt* viruses, including a clade 2 virus isolated from Indonesia in 2005. Two doses of the H5N1 *ca* vaccine provided complete protection from pulmonary replication in mice and ferrets and systemic dissemination of homologous and heterologous H5N1 *wt* viruses in mice. The high level of efficacy of the H5N1 1997 *ca* virus against challenge with antigenically and genetically diverse H5N1 *wt* viruses isolated over a span of 8 y suggests that the H5N1 viruses are evolving in nature to infect wild birds and domestic poultry, and not predominantly to evade antibodies as they do in humans. If the vaccine candidates described in this paper elicit a broadly cross-reactive protective immune response in humans, they would support the approach of developing one or two pandemic vaccine candidates for each subtype (H4 through H16) that are able to induce a broadly protective immune response to *wt* viruses within that subtype. The present findings serve to contradict recently expressed concerns about such a strategy [5,6].

All humans are immunologically naïve with respect to the HA at the start of a pandemic, and newly emerged pandemic viruses contain at least an antigenically novel HA and may also contain a novel NA subtype. Live, attenuated influenza virus vaccines potentially offer three major advantages over inactivated vaccines for rapid immunization of an immunologically naïve population [43]. First, in naïve populations, live virus vaccines induce higher levels of antibody than inactivated virus vaccines [21,44]. Second, live virus vaccines may induce protective immunity more quickly and would require fewer doses than inactivated vaccine [19]. In immunologically naïve populations, two doses of inactivated virus vaccine delivered 1 mo apart are required for induction of immunity [45], and significant immunity is not achieved until the response to the second dose is established, which occurs at about 40 d after immunization. Even at this time, mucosal immunity, especially that in the upper respiratory tract, is weak. In contrast, live influenza virus vaccines against human influenza viruses can induce a high level of immunity following a single dose, and immunity is achieved as early as 7–10 d following initial immunization, as indicated by the clearance of the vaccine virus from the respiratory tract. A live influenza virus vaccine induces both CD8^+^ T cell [46] and mucosal IgA antibodies in addition to serum antibodies [42], and therefore is the vaccine of choice for an immunologically naïve population. Third, the combined humoral and mucosal immune response generated by live virus vaccines in naïve populations results in broad protection against antigenically drifted strains. For example, the live, attenuated H3N2 human influenza vaccine containing an A/Wuhan/359/95 (H3N2)-like strain was effective in protecting against the A/Sydney/5/97 (H3N2)-like antigenic drift variant [47]. This may be a particularly useful feature in the event of a pandemic, in which a vaccine generated from the emergent pandemic virus strain is not available. Whether these theoretical advantages will be seen with live, attenuated H5N1 vaccines in humans remains to be established.

In summary, the modified H5N1 *ca* candidate vaccine viruses were immunogenic in mice, attenuated in mice, ferrets, and chickens, and protective in mice and ferrets against subsequent challenge with homologous and heterologous *wt* H5N1 influenza viruses. It is not known whether investigational live virus vaccines bearing avian influenza HA and NA genes in the AA *ca* background will be overattenuated in humans or will be associated with some residual virulence. Live, attenuated vaccines must be able to replicate to levels that elicit a protective immune response without causing disease in the host, so a balance between attenuation and immunogenicity must be achieved. A live, attenuated H5N1 vaccine would be administered to the general population only if an influenza pandemic caused by a virus of the H5N1 subtype were imminent, with confirmation of human-to-human transmission, or was already underway in the United States, and it would be used only on the recommendation of public health authorities. However, it is important to generate and carefully evaluate candidate live, attenuated H5N1 vaccines in clinical trials in humans because of their potential advantages over other vaccine approaches.

## Supporting Information

Table S1 Assessment of Pathogenicity and Infectivity of H5N1 *ca* Viruses in Chickens (38 KB DOC)

## Abbreviations

AA: influenza A/Ann Arbor/6/60
att: attenuation
BSL: biosafety level
ca: cold-adapted
CEF: chick embryo fibroblast
CPE: cytopathic effect
EID_50_: 50% egg infectious dose
HA: hemagglutinin
HAI: hemagglutination inhibition
HPAI: highly pathogenic avian influenza
LD_50_: 50% lethal dose
MDCK: Madin-Darby canine kidney
NA: neuraminidase
p.i.: postinfection
pfu: plaque-forming unit(s)
SE: standard error
SPF: specific pathogen-free
TCID_50_: 50% tissue culture infectious dose
ts: temperature sensitive
wt: wild-type

## Note Added in Proof

Reference 48 was added after this article was already in proof.

## References

[pmed-0030360-b001] de Jong MD, Tran TT, Truong HK, Vo MH, Smith GJ (2005). Oseltamivir resistance during treatment of influenza A (H5N1) infection. N Engl J Med.

[pmed-0030360-b002] Le QM, Kiso M, Someya K, Sakai YT, Nguyen TH (2005). Avian flu: Isolation of drug-resistant H5N1 virus. Nature.

[pmed-0030360-b003] Puthavathana P, Auewarakul P, Charoenying PC, Sangsiriwut K, Pooruk P (2005). Molecular characterization of the complete genome of human influenza H5N1 virus isolates from Thailand. J Gen Virol.

[pmed-0030360-b004] WHO Global Influenza Program Surveillance Network (2005). Evolution of H5N1 avian influenza viruses in Asia. Emerg Infect Dis.

[pmed-0030360-b005] Chen H, Smith GJ, Li KS, Wang J, Fan XH (2006). Establishment of multiple sublineages of H5N1 influenza virus in Asia: Implications for pandemic control. Proc Natl Acad Sci U S A.

[pmed-0030360-b006] Poland GA (2006). Vaccines against avian influenza—A race against time. N Engl J Med.

[pmed-0030360-b007] Nicholson KG, Colegate AE, Podda A, Stephenson I, Wood J (2001). Safety and antigenicity of non-adjuvanted and MF59-adjuvanted influenza A/Duck/Singapore/97 (H5N3) vaccine: A randomised trial of two potential vaccines against H5N1 influenza. Lancet.

[pmed-0030360-b008] Treanor JJ, Wilkinson BE, Masseoud F, Hu-Primmer J, Battaglia R (2001). Safety and immunogenicity of a recombinant hemagglutinin vaccine for H5 influenza in humans. Vaccine.

[pmed-0030360-b009] Gao W, Soloff AC, Lu X, Montecalvo A, Nguyen DC (2006). Protection of mice and poultry from lethal H5N1 avian influenza virus through adenovirus-based immunization. J Virol.

[pmed-0030360-b010] Hoelscher MA, Garg S, Bangari DS, Belser JA, Lu X (2006). Development of adenoviral-vector-based pandemic influenza vaccine against antigenically distinct human H5N1 strains in mice. Lancet.

[pmed-0030360-b011] Subbarao K, Chen H, Swayne D, Mingay L, Fodor E (2003). Evaluation of a genetically modified reassortant H5N1 influenza A virus vaccine candidate generated by plasmid-based reverse genetics. Virology.

[pmed-0030360-b012] Webby RJ, Perez DR, Coleman JS, Guan Y, Knight JH (2004). Responsiveness to a pandemic alert: Use of reverse genetics for rapid development of influenza vaccines. Lancet.

[pmed-0030360-b013] Stephenson I, Nicholson KG, Colegate A, Podda A, Wood J (2003). Boosting immunity to influenza H5N1 with MF59-adjuvanted H5N3 A/Duck/Singapore/97 vaccine in a primed human population. Vaccine.

[pmed-0030360-b014] Stephenson I, Bugarini R, Nicholson KG, Podda A, Wood JM (2005). Cross-reactivity to highly pathogenic avian influenza H5N1 viruses after vaccination with nonadjuvanted and MF59-adjuvanted Influenza A/Duck/Singapore/97 (H5N3) Vaccine: A potential priming strategy. J Infect Dis.

[pmed-0030360-b015] Li S, Liu C, Klimov A, Subbarao K, Perdue ML (1999). Recombinant influenza A virus vaccines for the pathogenic human A/Hong Kong/97 (H5N1) viruses. J Infect Dis.

[pmed-0030360-b016] Lipatov AS, Webby RJ, Govorkova EA, Krauss S, Webster RG (2005). Efficacy of H5 influenza vaccines produced by reverse genetics in a lethal mouse model. J Infect Dis.

[pmed-0030360-b017] Treanor JJ, Campbell JD, Zangwill KM, Rowe T, Wolff M (2006). Safety and immunogenicity of an inactivated subvirion influenza A (H5N1) vaccine. N Engl J Med.

[pmed-0030360-b018] Murphy BR (1993). Use of live attenuated cold-adapted influenza reassortant virus vaccines in infants, children, young adults and elderly adults. Infect Dis Clin Pract.

[pmed-0030360-b019] Murphy BR, Coelingh K (2002). Principles underlying the development and use of live attenuated cold-adapted influenza A and B virus vaccines. Viral Immunol.

[pmed-0030360-b020] Belshe RB, Gruber WC (2000). Prevention of otitis media in children with live attenuated influenza vaccine given intranasally. Pediatr Infect Dis J.

[pmed-0030360-b021] Mendelman PM, Rappaport R, Cho I, Block S, Gruber W (2004). Live attenuated influenza vaccine induces cross-reactive antibody responses in children against an A/Fujian/411/2002-like H3N2 antigenic variant strain. Pediatr Infect Dis J.

[pmed-0030360-b022] Reed LJ, Muench H (1938). A simple method of estimating fifty percent endpoints. Am J Hyg.

[pmed-0030360-b023] Hoffmann E, Neumann G, Kawaoka Y, Hobom G, Webster RG (2000). A DNA transfection system for generation of influenza A virus from eight plasmids. Proc Natl Acad Sci U S A.

[pmed-0030360-b024] United States Animal Health Association (1994). Report of the Committee on Transmissible Diseases of Poultry and Other Avian Species. Criteria for determining that an AI virus isolation causing an outbreak must be considered for eradication.

[pmed-0030360-b025] Stephenson I, Wood JM, Nicholson KG, Charlett A, Zambon MC (2004). Detection of anti-H5 responses in human sera by HI using horse erythrocytes following MF59-adjuvanted influenza A/Duck/Singapore/97 vaccine. Virus Res.

[pmed-0030360-b026] Jin H, Lu B, Zhou H, Ma C, Zhao J (2003). Multiple amino acid residues confer temperature sensitivity to human influenza virus vaccine strains (FluMist) derived from cold-adapted A/Ann Arbor/6/60. Virology.

[pmed-0030360-b027] Jin H, Zhou H, Lu B, Kemble G (2004). Imparting temperature sensitivity and attenuation in ferrets to A/Puerto Rico/8/34 influenza virus by transferring the genetic signature for temperature sensitivity from cold-adapted A/Ann Arbor/6/60. J Virol.

[pmed-0030360-b028] Bosch FX, Garten W, Klenk HD, Rott R (1981). Proteolytic cleavage of influenza virus hemagglutinins: Primary structure of the connecting peptide between HA1 and HA2 determines proteolytic cleavability and pathogenicity of avian influenza viruses. Virology.

[pmed-0030360-b029] Webster RG, Rott R (1987). Influenza virus A pathogenicity: The pivotal role of hemagglutinin. Cell.

[pmed-0030360-b030] Perdue ML, Garcia M, Senne D, Fraire M (1997). Virulence-associated sequence duplication at the hemagglutinin cleavage site of avian influenza viruses. Virus Res.

[pmed-0030360-b031] Maassab HF, DeBorde DC (1985). Development and characterization of cold-adapted viruses for use as live virus vaccines. Vaccine.

[pmed-0030360-b032] Chen H, Matsuoka Y, Swayne D, Chen Q, Cox NJ (2003). Generation and characterization of a cold-adapted influenza A H9N2 reassortant as a live pandemic influenza virus vaccine candidate. Vaccine.

[pmed-0030360-b033] Maines TR, Lu XH, Erb SM, Edwards L, Guarner J (2005). Avian influenza (H5N1) viruses isolated from humans in Asia in 2004 exhibit increased virulence in mammals. J Virol.

[pmed-0030360-b034] Lu X, Tumpey TM, Morken T, Zaki SR, Cox NJ (1999). A mouse model for the evaluation of pathogenesis and immunity to influenza A (H5N1) viruses isolated from humans. J Virol.

[pmed-0030360-b035] Gao P, Watanabe S, Ito T, Goto H, Wells K (1999). Biological heterogeneity, including systemic replication in mice, of H5N1 influenza A virus isolates from humans in Hong Kong. J Virol.

[pmed-0030360-b036] Bright RA, Cho DS, Rowe T, Katz JM (2003). Mechanisms of pathogenicity of influenza A (H5N1) viruses in mice. Avian Dis.

[pmed-0030360-b037] Lipatov AS, Krauss S, Guan Y, Peiris M, Rehg JE (2003). Neurovirulence in mice of H5N1 influenza virus genotypes isolated from Hong Kong poultry in 2001. J Virol.

[pmed-0030360-b038] Rowe T, Cho DS, Bright RA, Zitzow LA, Katz JM (2003). Neurological manifestations of avian influenza viruses in mammals. Avian Dis.

[pmed-0030360-b039] Hoffmann E, Lipatov AS, Webby RJ, Govorkova EA, Webster RG (2005). Role of specific hemagglutinin amino acids in the immunogenicity and protection of H5N1 influenza virus vaccines. Proc Natl Acad Sci U S A.

[pmed-0030360-b040] Neumann G, Watanabe T, Ito H, Watanabe S, Goto H (1999). Generation of influenza A viruses entirely from cloned cDNAs. Proc Natl Acad Sci U S A.

[pmed-0030360-b041] Fodor E, Devenish L, Engelhardt OG, Palese P, Brownlee GG (1999). Rescue of influenza A virus from recombinant DNA. J Virol.

[pmed-0030360-b042] Clements ML, Murphy BR (1986). Development and persistence of local and systemic antibody responses in adults given live attenuated or inactivated influenza A virus vaccine. J Clin Microbiol.

[pmed-0030360-b043] Subbarao K, Murphy BR, Fauci AS (2006). Development of effective vaccines against pandemic influenza. Immunity.

[pmed-0030360-b044] Johnson PR, Feldman S, Thompson JM, Mahoney JD, Wright PF (1986). Immunity to influenza A virus infection in young children: A comparison of natural infection, live cold-adapted vaccine, and inactivated vaccine. J Infect Dis.

[pmed-0030360-b045] Parkman PD, Hopps HE, Rastogi SC, Meyer HM (1977). Summary of clinical trials of influenza virus vaccines in adults. J Infect Dis.

[pmed-0030360-b046] Gorse GJ, O'Connor TZ, Newman FK, Mandava MD, Mendelman PM (2004). Immunity to influenza in older adults with chronic obstructive pulmonary disease. J Infect Dis.

[pmed-0030360-b047] Belshe RB, Gruber WC, Mendelman PM, Cho I, Reisinger K (2000). Efficacy of vaccination with live attenuated, cold-adapted, trivalent, intranasal influenza virus vaccine against a variant (A/Sydney) not contained in the vaccine. J Pediatr.

[pmed-0030360-b048] Steinhauer DA (1999). Role of hemagglutinin for the pathogenicity of influenza virus. Virology.

