# Supplementary material for: Live, Attenuated Influenza A H5N1 Candidate Vaccines Provide Broad Cross-Protection in Mice and Ferrets
Source: PLoS Med. 2006 Sep 12;3(9):e360. doi: 10.1371/journal.pmed.0030360 (PMC1564176; doi:10.1371/journal.pmed.0030360)
Supplement: Supplementary file 1 [file pmed.0030360.st001.doc]

TABLE S1. Assessment of pathogenicity and infectivity of H5N1 *ca* viruses in chickens

|  | Intravenous administration^a^ | | |  |  | Intranasal administration^b^ | | | | |  |
| --- | --- | --- | --- | --- | --- | --- | --- | --- | --- | --- | --- |
| Virus | Mortality  (survivors/  total) | | Mean time to death |  |  | Virus isolation from swabs | | | | | Antibody detected/total |
|  | | | | | | Oropharyngeal | |  | Cloacal | |  |
|  |  |  |  |  |  | # shedding/total | Viral titer^c^ |  | # shedding/total | Viral titer |  |
| H5N1 1997 *wt* | 0/8 | 1 day | |  |  | 8/8 | 6.3 |  | 8/8 | 5.9 | NA^d^ |
| H5N1 1997 *ca* | 8/8 | - | |  |  | 0/8 | ≤ 0.9 |  | 0/8 | ≤ 0.9 | 0/8 |
| H5N1 2003 *wt* | 0/8 | 1 day | |  |  | 8/8 | 7.4 |  | 8/8 | 4.5 | NA^d^ |
| H5N1 2003 *ca* | 8/8 | - | |  |  | 0/8 | ≤ 0.9 |  | 0/8 | ≤ 0.9 | 0/8 |
| H5N1 2004 *wt* | 0/8 | 1 day | |  |  | 8/8 | 6.4 |  | 8/8 | 5.8 | NA^d^ |
| H5N1 2004 *ca* | 8/8 | - | |  |  | 0/8 | ≤ 0.9 |  | 0/8 | ≤ 0.9 | 0/8 |

^a^Groups of eight 4-week old SPF chickens were inoculated i.v. with a 1:10 dilution of infectious allantoic fluid containing each virus, according to the standard intravenous pathogenicity test.

^b^10^6^ EID_50_ of virus administered intranasally in a volume of 0.1 mL.

^c^Oropharyngeal and cloacal swabs were collected for virus titration on day 3 after inoculation, or on day of death. Virus titers are expressed as log_10_ mean 50% egg infectious dose (EID_50_)/mL from 8 chickens. Virus titers from oropharyngeal and cloacal swabs from which virus was not isolated were assigned a numeric value of 0.9 for calculations.

^d^NA = not applicable: all chickens inoculated i.n. with H5N1 *wt* viruses died by day 3 after inoculation.
